# Supplementary material for: The Germinal Origin of Salivary and Lacrimal Glands and the Contributions of Neural Crest Cell-Derived Epithelium to Tissue Regeneration
Source: Int J Mol Sci. 2023 Sep 5;24(18):13692. doi: 10.3390/ijms241813692 (PMC10531458; doi:10.3390/ijms241813692)
Supplement: Supplementary file 1 [file ijms-24-13692-s001.zip › caption movie.pdf]

Movie S1. Three-dimensional (3D) images of axial section and coronal section of E13.5 TRiCK mouse.

Movie S2. Three colors of fluorescence expression of Trick mouse

Movie S3. 3D image of permeabilized duct
