# Supplementary material for: The Germinal Origin of Salivary and Lacrimal Glands and the Contributions of Neural Crest Cell-Derived Epithelium to Tissue Regeneration
Source: Int J Mol Sci. 2023 Sep 5;24(18):13692. doi: 10.3390/ijms241813692 (PMC10531458; doi:10.3390/ijms241813692)
Supplement: Supplementary file 1 [file ijms-24-13692-s001.zip › ijms-2566892-supplementary figures.pdf]

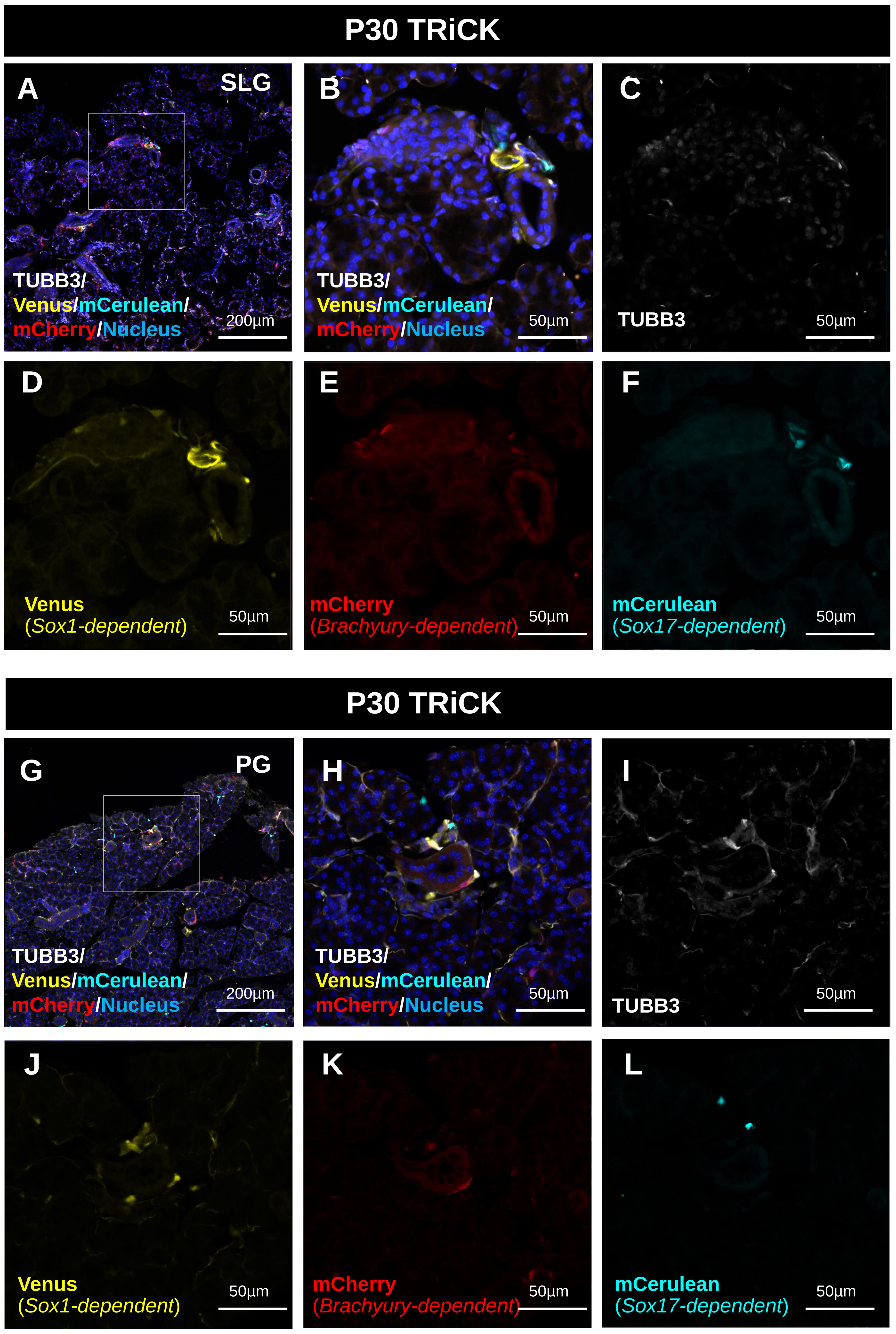

Figure S1. *Confocal microscopic images of TRiCK mouse sublingual gland (SLG) and parotid gland (PG).* (A-F). Boxed area in (A) is enlarged in (B-F). Scale bars: 200 µm (G), 50 µm (B-F). (G-L) The white arrowhead shows that TUBB3 is not localized to Venus-positive PG cells. Boxed area in (G) is enlarged in (H-L). Scale bars: 200 µm (G), 50 µm (H-L).

Figure S1

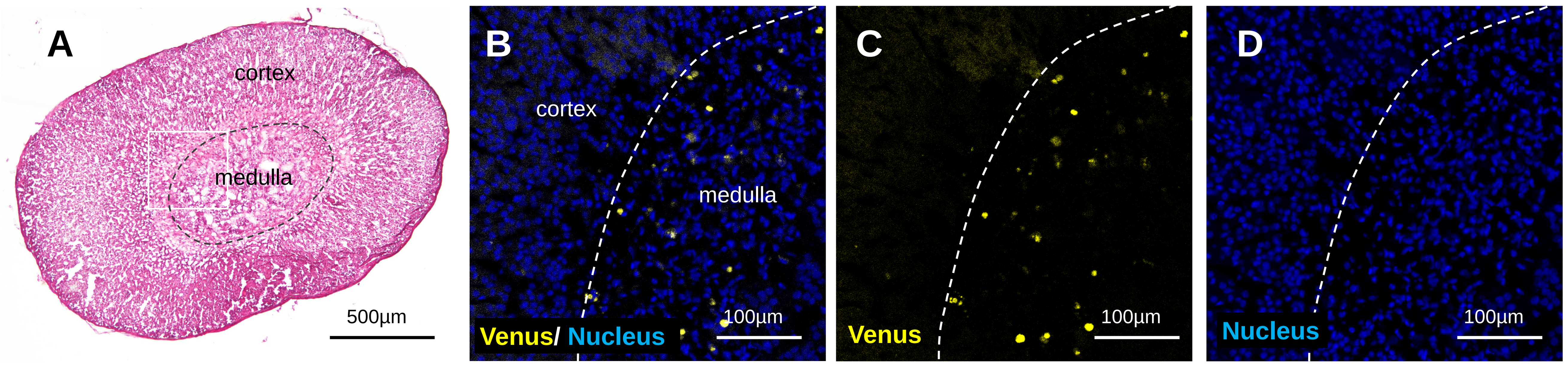

Figure S2. *Distribution of Sox1-dependent Venus positive cells in the TRiCK mouse adrenal gland.* (A-D) Mouse adrenal gland histology. Boxed area in (A) is enlarged in (B-D). co, cortex; me, medulla. Sox1-dependent Venus positive cells are dispersed in the medulla. Scale bars: 500 µm (A), 100 µm (B-D)

Figure S2

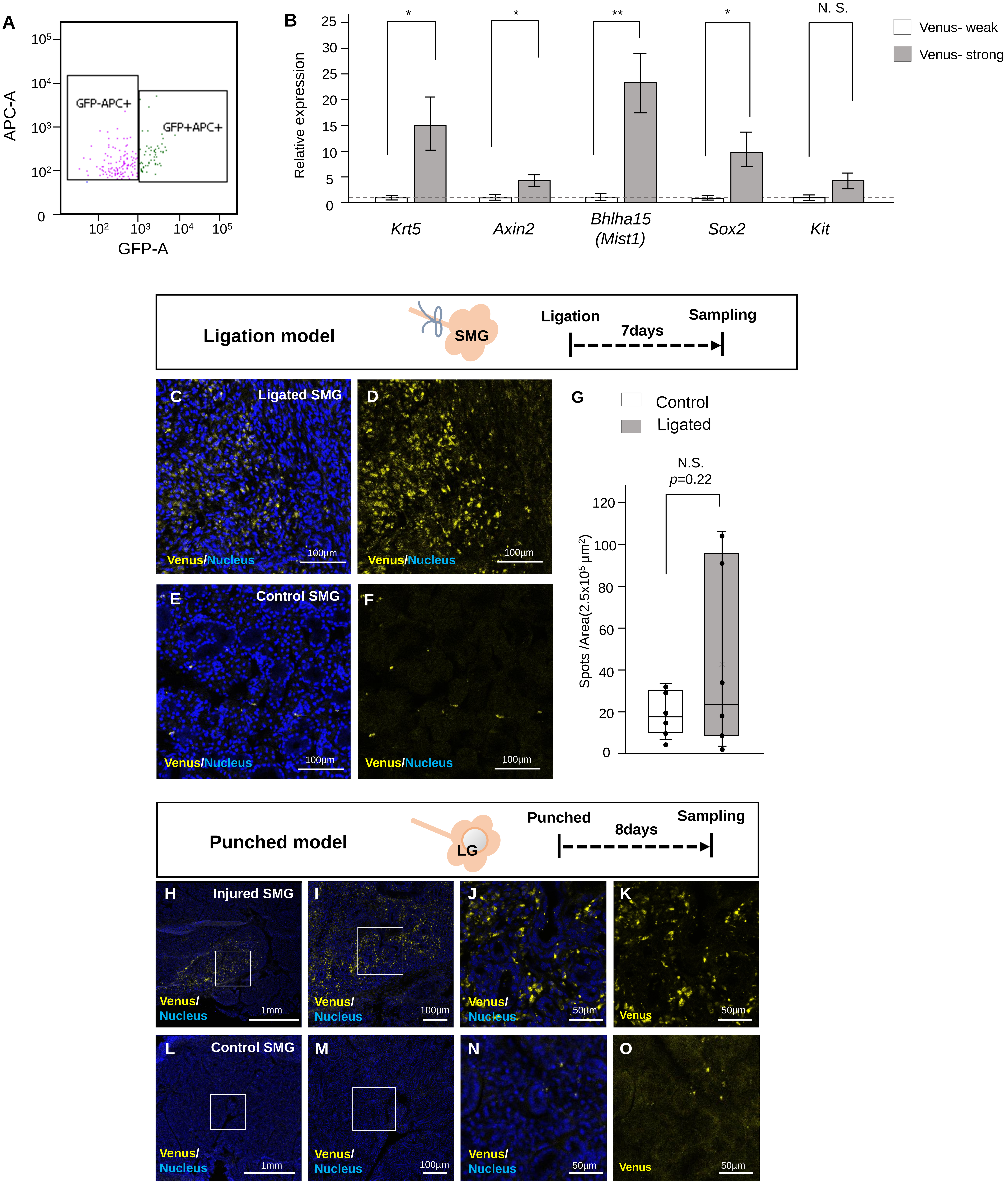
A
APC-A
GFP-A
GFP-APC+
GFP+APC+
B
Relative expression
Venus- weak
Venus- strong
*
**
N. S.
Krt5
Axin2
Bhlha15
(Mist1)
Sox2
Kit
Ligation model
SMG
Ligation
7days
Sampling
C
Ligated SMG
D
E
Control SMG
F
Venus/Nucleus
100µm
G
Control
Ligated
N.S.
p=0.22
Spots /Area(2.5x10⁵ µm²)
Punched model
LG
Punched
8days
Sampling
H
Injured SMG
I
J
K
L
Control SMG
M
N
O
Venus/
Nucleus
Venus
1mm
100µm
50µm

Figure S3

Figure S3. *Contribution of Sox1-dependent Venus-positive epithelial cells to SMG tissue regeneration.*
(A) Single cells isolated from TRiCK SMG were stained with CD44. CD44-positive SMG epithelial cells separated those with strong and weak expression of Venus by fluorescence-activated cell sorter. (B) Comparison of *CK5*, *Axin2*, Bhlha15(*Mist1*), *Sox1*, and *Kit* expression between Venus-high and Venus-low SMG cells by quantitative RT-PCR. Error bars indicate standard deviations. n=6 ** p<0.01, * p<0.05. Fluorescence images of TRiCK ligated (C, D) and control (E, F) SMG 7 days after ligation. Scale bars: 100 μm. (G) The number of Venus-positive cells in one area from six individual mice, showing that ligated salivary glands had higher numbers of Venus-positive cells. Bars indicate mean values and standard deviations. No significant (N. S.) differences were found between the two groups. (H-I) Fluorescence images of SMG at 8 days after foundation, (L-O) and its control side SMG. Boxed areas in (H, L) are enlarged in (H, L), respectively. Boxed areas in (I, M) are enlarged in (J, K) and (N,), respectively. Scale bars: 1 mm (H, L), 100 μm (I-K), 50μm (M-O).

Figure S3

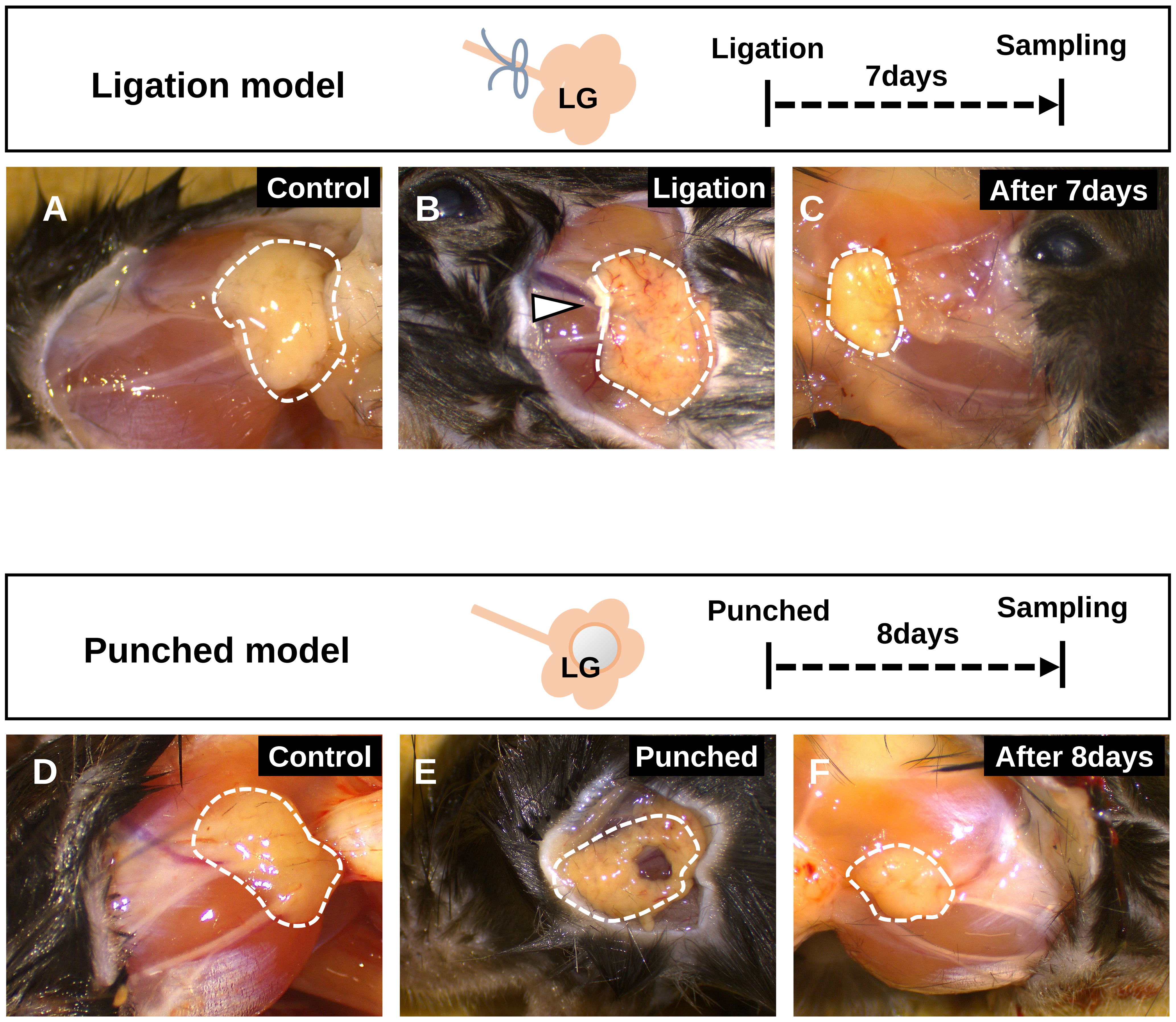

Figure S4. *Stereoscopic images of pre- and postoperative lacrimal glands.*
(A) Contralateral control gland of a ligated mouse. (B) Just after ligation and (C) 7 days after conduit ligation. White arrowhead in (B) points to the ligature area. LG is in the dotted area. (D) Contralateral control gland of the punched mouse. (E) Just after punching and (F) 8 days after the operation.

Figure S4

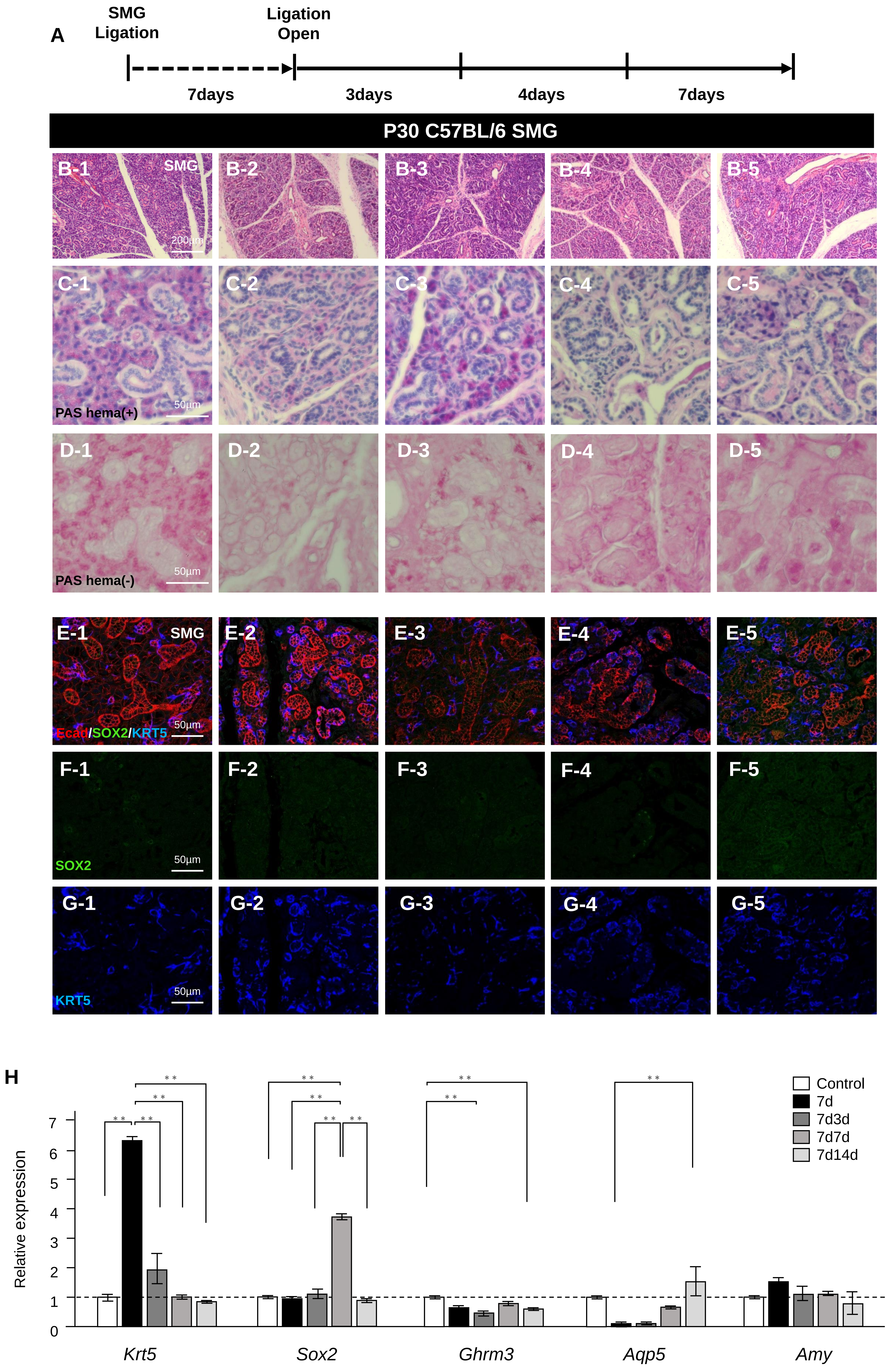
A
SMG Ligation
Ligation Open
7days
3days
4days
7days
P30 C57BL/6 SMG
B-1
SMG
200μm
B-2
B-3
B-4
B-5
C-1
PAS hema(+)
50μm
C-2
C-3
C-4
C-5
D-1
PAS hema(-)
50μm
D-2
D-3
D-4
D-5
E-1
SMG
Ecad/SOX2/KRT5
50μm
E-2
E-3
E-4
E-5
F-1
SOX2
50μm
F-2
F-3
F-4
F-5
G-1
KRT5
50μm
G-2
G-3
G-4
G-5
H
Relative expression
0
1
2
3
4
5
6
7
Krt5
Sox2
Ghrm3
Aqp5
Amy
Control
7d
7d3d
7d7d
7d14d
**

Figure S5

Figure S5. *SMG changes in a duct ligation mouse model.*

(A) Time course of sampling in a duct ligation model. Images before ligation of SMG (B-1 to G-1). Images of ligated SMG obtained at 7 days after ligation (B-2 to G-2), at 3 days (B-3 to G-3), at 7 days (B-4 to G-4), or at 14 days (B-5 to G-5) after removal of the ligature. The process of SMG regeneration is shown with HE staining (B), hematoxylin and PAS staining (C), and PAS staining only (D). (E-G) Confocal images of protein expression obtained during salivary gland regeneration. E-cadherin (E-cad) (red), SOX2 (green), Cytokeratin 5 (CK5) (blue). Scale bars: 200 μm (B), 50 μm (C-G). (H) Changes in gene expression of salivary gland markers and regeneration markers as revealed by real time RT-PCR. ** $p<0.01$, * $p<0.05$.

Figure S5

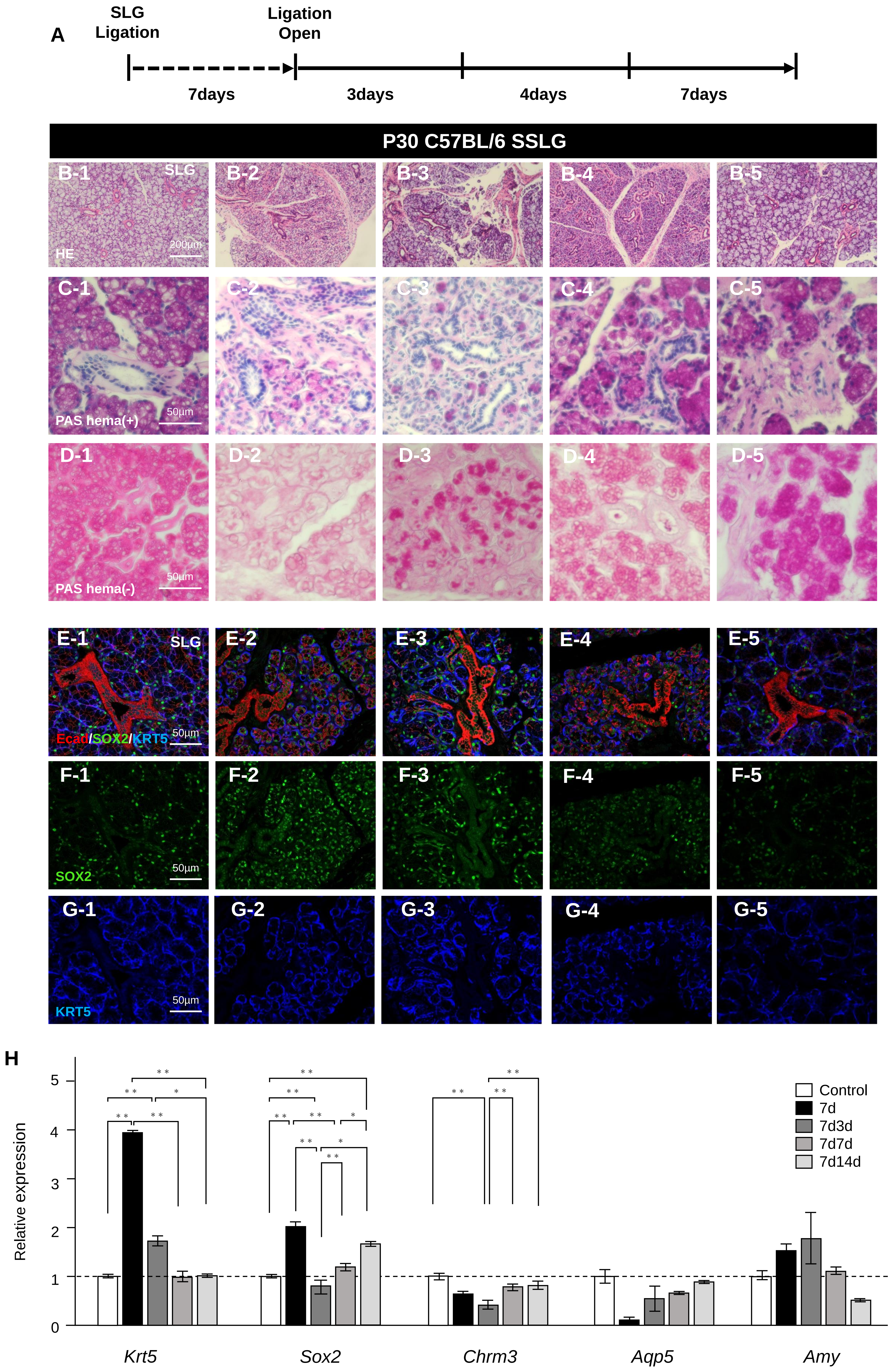
A
SLG Ligation
Ligation Open
7days
3days
4days
7days
P30 C57BL/6 SSLG
B-1
SLG
HE
200µm
B-2
B-3
B-4
B-5
C-1
PAS hema(+)
50µm
C-2
C-3
C-4
C-5
D-1
PAS hema(-)
50µm
D-2
D-3
D-4
D-5
E-1
SLG
Ecad/SOX2/KRT5
50µm
E-2
E-3
E-4
E-5
F-1
SOX2
50µm
F-2
F-3
F-4
F-5
G-1
KRT5
50µm
G-2
G-3
G-4
G-5
H
Relative expression
0
1
2
3
4
5
Control
7d
7d3d
7d7d
7d14d
Krt5
Sox2
Chrm3
Aqp5
Amy

Figure S6

Figure S6. *SLG changes in a duct ligation mouse model.*

(A)Time course of sampling in a duct ligation model. Images before ligation of SLG (B-1 to G-1). Images of ligated SLG obtained at 7 days after ligation (B-2 to G-2), at 3 days (B-3 to G-3), at 7 days (B-4 to G-4), or at 14 days (B-5 to G-5) after removal of the ligature. The process of SLG regeneration is shown with HE staining (B), hematoxylin and PAS staining (C), and PAS staining only (D). (E-G) Confocal images of protein expression obtained during salivary gland regeneration. E-cad (red), Sox2 (green), Cytokeratin 5 (CK5) (blue). Scale bars: 200 μm (B), 50 μm (C-G). (H) Changes in expression of salivary gland marker and regeneration marker genes as revealed by real time RT-PCR. ** $p<0.01$, * $p<0.05$.

Figure S6

Table S1. Primer sequences for real time qPCR	
Primer names	Primer sequences
Gapdh -F	5′ -CCATCACCATCTTCCAGGAG-3′
Gapdh -R	5′ -GCATGGACTGTGGTCATGAG-3′
Sox10 -F	5′ -CACATCGACTTCGGCAACGT-3′
Sox10 -R	5′ -CCGTTGGGTGGCAGGTATT-3′
Krt5 -F	5′ -ATCGCCACTTACCGCAAGCTGCTGGAGGG-3′
Krt5 -R	5′ -AAACACTGCTTGTGACAACAGAG-3′
Axin2 -F	5′ -AAGAGAAGCGACCCAGTCAATCC-3′
Axin2 -R	5′ -GGTTCCACAGGCGTCATCTCC-3′
Bhlha15(Mist1) -F	5′ -GGCTAAAGCTACGTGTCCTTG-3′
Bhlha15(Mist1) -R	5′ -GGTGAGGCCCTTCCAACT-3′
Sox2 -F	5′ -GGGAAATGGGAGGGGTGCAAAAGAGG-3′
Sox2 -R	5′ -TTGCGTGAGTGTGGATGGGATTGGTG-3′
Kit -F	5′ -CCCATGTATGAAGTACAGTGGAAG-3′
Kit -R	5′ -CTGATTAAGTCGGATGCGGCCATG-3′
Chrm3 -F	5′ -TCGGTAGAGCGGACTGGACA-3′
Chrm3 -R	5′ -TCCACTGAGCAAGTCAGAAGTGAAG-3′
Aqp5 -F	5′ -TGGAGCAGGCATCCTGTACT-3′
Aqp5 -R	5′ -CGTGGAGGAGAAGATGCAGA-3′
Amy -F	5′ -GGATGGAGAAAAGATGTCCTAC-3′
Amy -R	5′ -CATCACCCGTGTGAAACC-3′

Table S1

Table S2. Frequency of Venus-positive glandular epithelial cells																		
	SMG				SLG				PG				LG				Nervus opticus	
	Duct		Acinus		Duct		Acinus		Duct		Acinus		Duct		Acinus			
Sample	Right	Left	Right	Left	Right	Left	Right	Left	Right	Left	Right	Left	Right	Left	Right	Left	Right	Left
A	-	-	-	-	-	-	-	-	+	+	-	-	+	-	-	+	+	+
B	-	-	-	-	-	-	-	-	-	+	-	-	-	-	-	-	+	+
C	-	-	-	-	-	-	-	-	-	-	+	-	-	-	-	-	+	+
D	-	+	-	+	-	-	-	-	+	+	-	-	-	+	-		+	+
E	-	-	-	-	-	-	-	-	+	+	-	-	+	-	+	+	+	+
F	-	-	-	-	-	-	-	-	+	+	-	-	-	+	-	-	+	+
G	+	+	-	-	+	-	+	-	+	+	-	-	+	+	+	-	+	+
H	-	+	-	-	-	+	-	-	-	+	-	-	-	+	-	-	+	+
I	+	-	-	-	-	-	-	-	+	-	-	-	+	-	+	-	+	+
J	+	-	-	-	+	-	-	+	+	-	-	-	-	-	-	-	+	+
K	+	+	-	-	+	-	-	-	+	+	-	-	-	-	-	-	+	+
Percentage of individuals with positive epithelial appearance organ	36.36%		4.54%		18.18%		9.09%		72.72%		4.54%		36.36%		22.72%		100%	

Table S2. *Frequency of Venus-positive glandular epithelial cells.*

Table S2
